# EBA (Engaged but Amotivated) in AI-enhanced EFL learning: a qualitative study from a Chinese higher vocational context

**DOI:** 10.3389/fpsyg.2025.1643653

**Published:** 2025-09-09

**Authors:** Lei Cao, Amelia Abdullah

**Affiliations:** ^1^Department of Foreign Languages Teaching, Wuxi University of Technology, Wuxi, China; ^2^School of Educational Studies, Universiti Sains Malaysia, Penang, Malaysia

**Keywords:** GenAI, EBA, HVC, EFL, self-determination theory, engagement, qualitative study

## Abstract

**Introduction:**

The integration of generative artificial intelligence (GenAI) into English as a Foreign Language (EFL) pedagogy entails both potentials and pitfalls. This study investigates a newly observed phenomenon: the “Engaged but Amotivated” (EBA) learners, who demonstrate behavioral participation yet experience a profound lack of motivation. Grounded in Self-Determination Theory (SDT) and multidimensional engagement framework, the study investigates how GenAI tools subtly influence EFL learners’ motivation and engagement, particularly in low-proficiency vocational contexts.

**Methods:**

This study adopted a qualitative research design within a Chinese higher vocational college, spanning two academic semesters. A rich tapestry of data was meticulously gathered through immersive classroom observations, in-depth semi-structured interviews with 39 first-year EFL students, and trace-based learning management system logs. Thematic analysis was employed to identify nuanced patterns and emergent themes, illuminating the participants’ lived experiences and their intricate interactions with GenAI-enhanced EFL instruction.

**Results:**

The analysis identified three core themes defining the EBA learner dynamic: ① Performative participation: engagement as institutional compliance; ② Motivational stagnation: cognitive overload as an obstacle; and ③ Identity ambivalence: GenAI as enabler and eroder.

**Discussion:**

This study interrogates the prevailing assumption that visible engagement signifies meaningful learning, cautioning against an overreliance on behavioral indicators in AI-mediated instructional settings, particularly in low-proficiency contexts. It further challenges the widespread optimism surrounding AI’s purported motivational benefits. The findings yield critical implications for pedagogical design, AI system development, and teacher education—particularly within underexplored vocational education contexts.

## 1 Introduction

Over recent years, the integration of generative artificial intelligence (GenAI) into language education has significantly reshaped teaching and learning practices, particularly in the context of English language learning (Abed et al., 2024; Banun, 2025; Baskara, 2023; Fathi and Rahimi, 2024; Febriani, 2024; Fitria, 2023; Liando et al., 2025; Özdere, 2023). As a major global player, China has experienced rapid growth in its English language learner population, especially among English as a Foreign Language (EFL) learners. Concurrently, GenAI-based tools and applications—such as DeepSeek, Kimi, Doubao (豆包), Xuexitong (学习通), and ChatGPT—are being increasingly adopted and adapted in Chinese higher education as part of national initiatives to modernize education and advance pedagogies (Gong and Deng, 2024). These tools aim to personalize instruction, foster learner autonomy, enhance motivation, promote engagement, and ultimately improve learning outcomes (Ahimsa and Khawa, 2025; Kayaalp, 2025; Wei, 2023).

Existing scholarships highlight a growing fascination and critical focus on empowering EFL teaching and learning with AI in China (Jiang, 2022; Xu and Li, 2024), which can be systematically grouped into three key areas: pedagogical applications and learner psychology. First, the pedagogical application area focuses on how AI tools are integrated into instructional design, classroom practices, and learning outcomes. For instance, Luo et al. (2025) found that both AI-only and teacher-AI hybrid feedback improved EFL learners’ argumentative writing, with the hybrid model yielding more comprehensive results. Tang (2025) reported that university learners saw improvement in writing quality through AI, especially in terms of language refinement and strategy, while calling for pedagogical models that balance AI with creativity. Yang J. (2024) studied pre-service teachers’ use of AI translation tools, highlighting their usefulness but also the need for guidance to prevent overuse. Zou et al. (2023) emphasized the benefits of AI-assisted speaking practice through social networks. Meanwhile, Liu and Quan (2022) applied big data and speech modeling to create AI-driven pronunciation feedback that improves learners’ oral accuracy.

The psychological strand of research emphasizes learner motivation, engagement, and emotional responses in AI-enhanced environments. Yuan and Liu (2025) found that tools like Duolingo significantly increased learners’ engagement and enjoyment. Xu and Liu (2025) compared ChatGPT and Duolingo, showing both improved motivation, autonomy, and critical thinking. Liu et al. (2024a) highlighted learners’ positive attitudes toward AI in informal English learning. In follow-up studies, Liu and colleagues explored how AI use supports persistence and identity development in informal contexts (Liu et al., 2024b; Liu et al., 2025). These studies demonstrate how AI shapes both learning behaviors and emotional investment. Wang X. et al. (2025) found that classroom climate, resilience, and AI literacy strongly influence engagement, while teacher support remains crucial for motivation and emotional well-being (Shen et al., 2024).

Despite this progress, little is known about AI’s impact on learners in Chinese higher vocational colleges (HVCs), who often differ from university students in academic background, goals, and digital readiness. As Crompton et al. (2024) noted, there is “a need for future studies conducted in a wider range of geographies and contexts.” Understanding AI’s influence in lower-track education like HVCs is thus essential but underexplored.

This qualitative study investigates how AI-mediated instruction shapes EFL learners’ motivation and engagement in Chinese HVCs. While frequent interaction with AI tools may indicate behavioral engagement, scholars warn that participation alone does not confirm genuine motivation (Reeve and Tseng, 2011; Skinner, 2016). In light of this, the study proposes the construct of “Engaged but Amotivated” (EBA)—students who complete tasks and use AI tools but report low personal drive or affective connection. This concept questions the assumption that visible engagement equates to meaningful learning.

By offering a deeper view of learner engagement, this study contributes to the growing conversation on AI in EFL education. The EBA lens helps explore how extrinsic pressures and AI use intersect with learner agency. Focusing on the under-researched HVC context, the study fills a gap in AI-EFL literature. It also offers practical insights for teachers, curriculum developers, and AI designers, encouraging them to look beyond behavior and support more meaningful, sustained motivation. In doing so, the research answers Crompton et al.’s (2024) call to better understand the challenges that AI brings to language learning.

## 2 Literature review

The integration of GenAI into EFL pedagogy has led to significant shifts in instructional practices worldwide, with China emerging as a key site of AI-driven educational reform (Huang et al., 2025; Liu and Xiao, 2025; Pan and Wang, 2025; Tang, 2025; Wei Q. et al., 2025; Xu and Liu, 2025). AI-powered platforms are widely recognized for enhancing learner engagement, supporting language development, and offering personalized instruction (Hong, 2023; Huang and Tan, 2023; Liu and Wang, 2024; Luo et al., 2025). However, these advancements raise an important question: Does visible participation in AI-enhanced learning truly indicate genuine motivation? To explore this, the following review synthesizes three key strands of research: (1) motivational and engagement theories in EFL learning, (2) the emerging concept of the “Engaged but Amotivated” (EBA) learner in AI-mediated environments, and (3) the specific characteristics of Chinese HVC EFL learners. Despite representing over half of China’s tertiary student population (Ministry of Education of the People’s Republic of China, 2024), this group remains underrepresented in AI-enhanced EFL research, highlighting a gap in inclusive educational development.

### 2.1 Theoretical framework

The integration of AI-mediated instruction in EFL education requires a nuanced understanding of how motivation and behavior shape learner engagement. Central to this discussion is Self-Determination Theory (SDT) by Deci and Ryan (1985, 2000) and Ryan and Deci (2020), which remains a key framework for examining motivation in educational settings. According to SDT, motivation lies on a continuum from external to internal regulation and is influenced by the satisfaction of three basic psychological needs: autonomy, competence, and relatedness. Recent studies show that AI-enhanced environments can affect these motivational factors in English language learning (Ali et al., 2023; Aly et al., 2024; Ebadi and Amini, 2024; Fandiño et al., 2019; Hao et al., 2024; He, 2024; Madinabonu, 2024; Moybeka et al., 2023; Ramadhani, 2025; Silitonga et al., 2023; Suciati et al., 2024; Tran, 2024; Wang F. et al., 2025; Yang T., 2024; Yaşar and Karagücük, 2024).

However, frequent interaction with AI tools does not necessarily reflect internal motivation. To explore this further, Fredricks et al.’s (2004) tripartite model of engagement—covering behavioral, emotional, and cognitive dimensions—offers a valuable lens. While behavioral signs like task completion are easy to track, they may not reflect deeper learning or motivation, especially when AI tools lead to surface-level engagement. Although studies have shown AI’s positive effect on behavioral outcomes (Bhatt and Muduli, 2024; Ellikkal and Rajamohan, 2024), other findings suggest that overreliance on GenAI may weaken critical thinking and personal effort (Alasadi and Baiz, 2023; Pido et al., 2025). Waluyo and Kusumastuti (2024) also warn that high engagement with GenAI does not always lead to better academic results, further challenging the link between AI use and authentic learning gains.

This complexity calls for a more careful look at how GenAI shapes learners’ motivation and engagement. Reeve and Tseng (2011) and Skinner (2016) emphasize that externally driven behavior should not be mistaken for genuine commitment. In AI-supported EFL contexts, visible engagement may not indicate internal motivation, showing that behavioral data alone may be insufficient for understanding true learning progress.

### 2.2 “Engaged but Amotivated”: a new construct

To address this paradoxical phenomenon, the present study introduces the construct of the “Engaged but Amotivated” (EBA) learner. Drawing on Self-Determination Theory (Deci and Ryan, 1985, 2000; Ryan and Deci, 2020), EBA refers to students who actively participate in learning tasks—often driven by AI prompts or institutional expectations—yet report low intrinsic motivation, limited emotional connection, and even low academic performance. Specifically, EBA students demonstrate consistent behavioral participation—such as task completion, punctual submission, and frequent AI interaction—without exhibiting corresponding intrinsic or identified motivational regulation. In short, these learners are “doing” without genuinely “wanting.”

In AI-mediated environments, this disconnect between action and motivation is often obscured by the high visibility of behavioral participation. The gamification of tasks, automated feedback loops, and performance-tracking mechanisms commonly integrated in GenAI platforms may incentivize learners to complete tasks efficiently, but not necessarily meaningfully. According to Fredricks et al. (2004), true engagement involves not only behavioral indicators but also cognitive and emotional involvement. In the case of EBA learners, these latter dimensions often remain underdeveloped, with students demonstrating a passive orientation to tasks and limited evidence of deeper cognitive processing. Additionally, insights from cognitive load theory (Sweller, 2011) offer a complementary perspective. When low-proficiency learners overly depend on AI to generate content, their active involvement in the high intrinsic load and the overall cognitive process essential for language development may be diminished.

While the EBA construct is new, it overlaps with—but is also distinct from—existing ideas such as amotivation, surface learning, and performance-approach learning. Amotivation means a student has no motivation and usually does not take part in learning activities (Deci and Ryan, 1985, 2000; Ryan and Deci, 2020). In contrast, EBA students appear active and complete tasks but feel no real interest or purpose. EBA is harder to notice because the lack of motivation is hidden by visible participation. Surface learning refers to the learning with “motive of meet institutional requirements minimally, and the congruent strategy is limiting the target to essentials that may be reproduced through rote learning” (Biggs, 1988, p. 129). Although surface learning and EBA both show limited cognitive engagement, they differ in motivation and behavior. Surface learners are extrinsically motivated and focus on minimum requirements. In contrast, EBA learners appear highly active but lack meaningful motivation. Performance-approach learning emphasizes outcome-based goals (Elliot, 1999; Elliot and Moller, 2003; Elliot et al., 2005; Gilbert and Elliot, 2024) where learners are driven by grades or comparison, which can still involve high motivation, albeit extrinsic. In contrast, EBA uniquely combines high visible effort with an inner motivational void. It describes students who appear engaged but experience emotional detachment and a lack of personal meaning in their learning actions.

Recent studies support this pattern. Liu et al. (2024a) found that Chinese university learners using AI tools in informal digital learning often engage out of habit or utility rather than personal interest, particularly when autonomy is limited. Similarly, Pan and Wang (2025) and Zhou and Hou (2024) noted how AI’s efficiency and structure can undermine the human elements of teaching and learning, raising concerns about over-reliance and emotional disconnection. Shen et al. (2024) further argued that emotional support from teachers remains essential for fostering motivation and further engagement, suggesting that AI, by itself, cannot sustain learner motivational well-being.

In sum, the EBA construct draws attention to a hidden problem in AI-mediated education: the illusion of engagement. It reminds educators and designers that participation does not always equal meaningful learning. Clarifying this concept helps separate active behavior from authentic motivation, which is essential for responsible use of AI tools in EFL contexts.

### 2.3 Chinese higher vocational colleges: a critical context

Chinese HVCs represent a unique and often overlooked setting within the broader context of GenAI-enhanced EFL education. Enrolling over half of the nation’s tertiary students (Ministry of Education of the People’s Republic of China, 2024; Xu, 2025), these institutions primarily serve learners who are placed into vocational tracks through academic streaming systems such as the gaokao (高考) (Xiong, 2011). As a result, students in HVCs often begin their English studies with lower academic readiness, weaker language proficiency, and a more practical orientation toward language learning (Pan and Dapat, 2023; Wang, 2024; Wang and Chen, 2021). These characteristics position HVCs as a key context for understanding how GenAI affects motivation, learning behavior, and learner identity differently than in traditional university settings (Wang, 2024; Wei W. et al., 2025).

Despite national goals for inclusive digital transformation, HVC learners are frequently absent from both policy and empirical discussions. Most studies of AI-based EFL instruction in China focus on university students with stronger self-regulation, academic ambition, and digital literacy (Liu et al., 2025; Xu and Liu, 2025). In contrast, vocational learners may struggle with the self-directed learning and critical evaluation skills needed to use GenAI tools effectively. This makes them particularly susceptible to the EBA pattern, where task completion masks limited internal engagement.

The institutional environment adds further complexity. Curricula in HVCs are closely aligned with vocational training and often treat English as a skill to pass tests or secure employment rather than as a tool for personal or intellectual growth (Cao and Chen, 2023; Wei Q. et al., 2025; Wei W. et al., 2025). Within this framework, AI systems that reward efficiency may unintentionally encourage mechanical completion of tasks, rather than deeper processing or genuine interest. Students may depend on AI-generated content or aim for performance metrics without actively engaging in language development.

Sociocultural influences further shape learners’ attitudes. Many HVC students come from lower-income families and face pressure to prioritize immediate employability. Traditional values such as diligence and conformity (Cheng, 2020) may align with AI’s output-driven design, reinforcing behaviors aimed at meeting visible expectations rather than fostering reflective or self-directed learning. In this context, GenAI may be viewed as a functional but impersonal tool, especially if it challenges learners’ confidence or sense of relevance (Riser, 2025).

Given these dynamics, it is essential to examine GenAI use in HVCs through a nuanced, learner-centered lens. The question is not simply how to integrate technology, but how to align it with learners’ needs and realities. Without thoughtful pedagogical support, GenAI risks reinforcing superficial learning and widening existing gaps. Constructs like EBA can help identify misalignments between participation and meaningful engagement. A context-sensitive approach that fosters learner autonomy and emotional connection is key to ensuring that technology acts as an enabler rather than a barrier.

### 2.4 Research questions

How do AI tools influence the motivation of EFL learners in a Chinese vocational college?
How do EFL learners show engagement when using AI tools, and what does this reveal about their learning experiences?

## 3 Methodology

This study employs a qualitative study methodology using thematic analysis to investigate the issue of “Engaged but Amotivated” (EBA) learners in a GenAI-enhanced EFL classroom within a Chinese HVC. According to Denzin and Lincoln (2008), qualitative research involves systematically collecting diverse empirical materials—including case studies, interviews, and observations—to deeply examine routine and problematic moments and meanings in individuals’ lives. It is inherently reflective, interpretive, and descriptive, aiming to understand and represent human experiences from the perspective of participants within specific contexts (Aspers and Corte, 2019). The paradox of EBA—where learners demonstrate visible behavioral engagement while remaining motivationally detached—presents a particularly complex and context-sensitive educational challenge. In the Chinese vocational education setting, a qualitative approach is especially suitable, as it allows for a rich, complex exploration of students’ motivational and behavioral landscapes. This methodology enables the capture of learners’ subjective experiences, illuminating the tensions and contradictions that define EBA, and offering insights into their behaviors and perceptions shaped by the educational and cultural context.

As Clarke and Braun (2017, p. 297) assert, thematic analysis “is unusual in the canon of qualitative analytic approaches, because it offers a method—a tool or technique, unbounded by theoretical commitments—rather than a methodology (a theoretically informed and confined framework for research).” Due to this distinctive theoretical flexibility, thematic analysis is particularly suitable for the present qualitative study, as it allows for the exploration and development of the novel theoretical construct of “EBA.” This flexibility enables the integration and examination of existing theoretical frameworks, such as Self-Determination Theory (SDT) and Fredricks et al.’s engagement model, while simultaneously providing space for inductively deriving new theoretical insights from participants’ lived experiences. Consequently, thematic analysis effectively supports the multifaceted exploration of EBA, facilitating a rich understanding of the complex dynamics shaping learners’ motivation and engagement within GenAI-mediated EFL environments.

### 3.1 Participants

**Figure 1:**
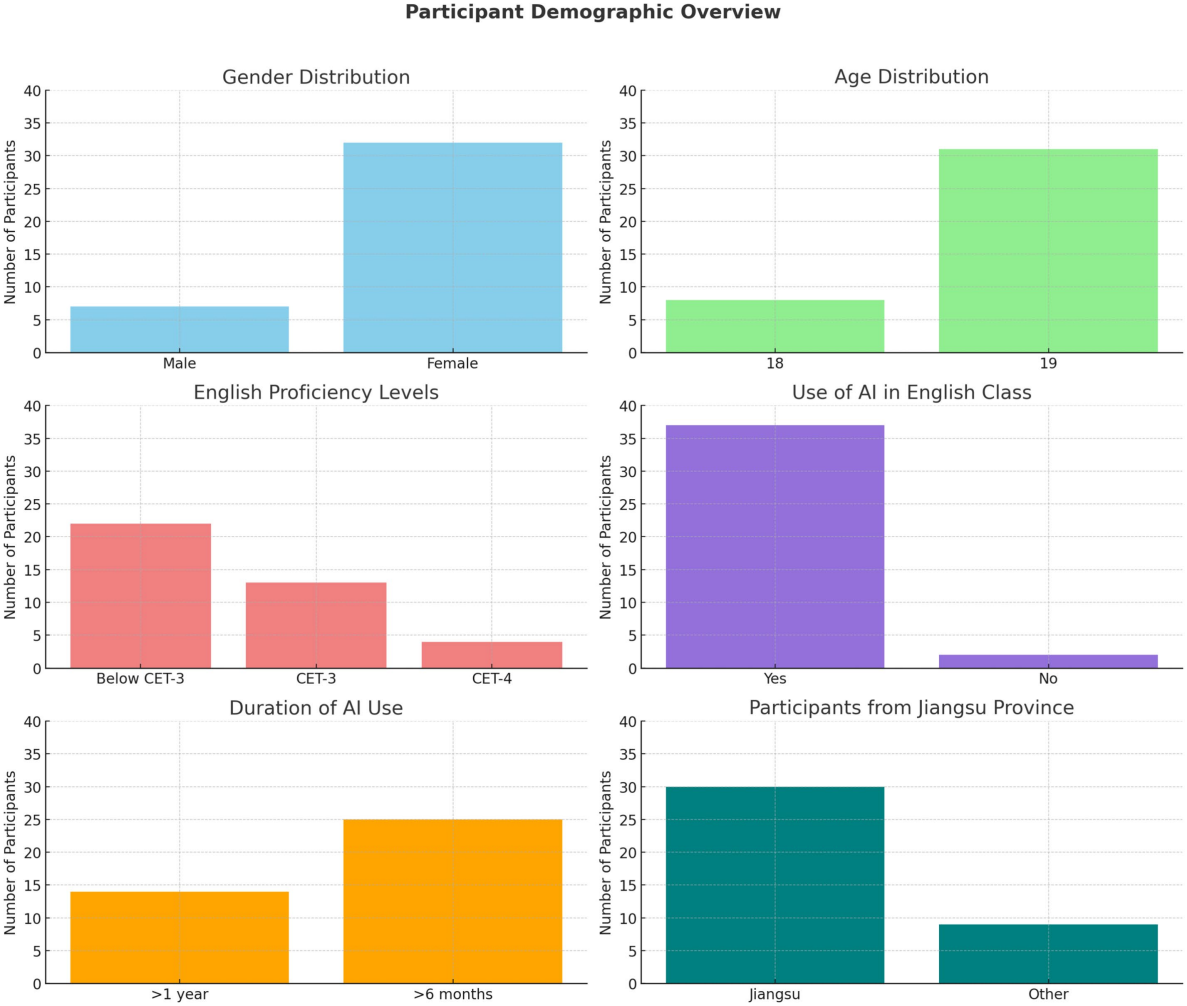
Participant demographic overview.

The study was conducted at a first-year class at an HVC located in eastern China. Firstly, located in the Yangtze River Delta, a region recognized as the most economically and technologically advanced in China, this college was purposefully selected due to its proactive incorporation of GenAI tools into first-year EFL classes as part of a broader digital education reform initiative. Participants were selected through purposive sampling, targeting students actively engaged in GenAI-mediated English classes but exhibiting varied motivational profiles. The final sample included 39 first-year students majoring in Digital Media, chosen for their consistent exposure to GenAI tools—such as Xuexitong, Doubao(豆包), DeepSeek, and Kimi—in class and for assignments (Figure 1).

The participant group was predominantly female and aged 19, reflecting the typical profile of first-year students in vocational EFL programs. Most students (89.74%) reported English proficiency at or below CET-3, which roughly corresponds to the A2 to low B1 levels on the Common European Framework of Reference for Languages (CEFR). This indicates limited language competence, with learners capable of basic communication but often struggling with academic English—likely influencing their reliance on GenAI tools and contributing to patterns of extrinsically driven engagement. Nearly all participants had prior experience using GenAI tools in class, with over 60% using them for more than 6 months. A majority (76.9%) were from Jiangsu Province, providing a relatively consistent regional context.

### 3.2 Data collection

Data collection spanned two academic semesters (approximately 36 weeks), providing longitudinal insights into learners’ engagement and motivational dynamics within consistent instructional settings.

#### 3.2.1 Classroom observations

Weekly non-participant observations were conducted during 12 class sessions. Observations focused on:

1. Student interaction with GenAI tools.
2. Task completion patterns.
3. Indicators of emotional engagement or disaffection.
4. Collaborative behaviors and active participation.

Field notes documented behaviors indicative of the EBA condition, such as sustained task focus but limited emotional and verbal involvement, guided by Fredricks et al.’s (2004) engagement framework.

#### 3.2.2 Semi-structured interviews

To collect detailed information about students’ experiences in AI-supported English learning, semi-structured interviews were conducted at the end of the second semester. This timing was chosen to ensure that participants had completed the full EFL course and had enough experience with AI tools to reflect meaningfully on their learning.

A total of 39 first-year students took part in one-on-one interviews. Each session lasted about 30 min. The interview questions were designed to explore students’ learning backgrounds, use of AI tools, engagement patterns, and their views on motivation and learner identity.

The protocol was first piloted with four students who shared similar characteristics with the main sample. Based on their responses, minor adjustments were made to enhance clarity and flow. Additional prompts in Chinese were incorporated to support ease of communication and promote more natural interaction.

All interviews were conducted in a quiet and private setting to reduce distractions. Mandarin Chinese served as the primary interview language, while English was used occasionally, especially when referring to specific tools or concepts. This bilingual approach supported accurate expression and comfort for participants. Interviews were audio-recorded with prior consent and conducted in line with ethical standards approved by the host institution. Participants were assured of confidentiality and reminded that they could withdraw at any point. All recordings were transcribed and securely stored for analysis, which is explained in Section 3.3.

#### 3.2.3 LMS log analysis

Learning Management System (LMS) data were retrieved from Xuexitong (学习通), an AI-enhanced digital platform that served as the primary medium for teaching, learning, and assignment management throughout the two-semester EFL course. The platform automatically recorded a range of learner interactions, including: frequency and duration of engagement with generative AI tools; submission and resubmission behaviors; engagement with AI-generated feedback and revision suggestions; time-on-task metrics.

These AI-supported learning traces were leveraged to triangulate and reinforce qualitative findings from interviews and classroom observations. In particular, the LMS logs were instrumental in identifying participants who demonstrated sustained behavioral engagement with the platform yet expressed signs of motivational detachment during reflective interviews—an indicative pattern within the Engaged but Amotivated (EBA) profile.

### 3.3 Data analysis

To obtain a comprehensive understanding of the participants’ experiences with AI-mediated English instruction, qualitative data from interviews, classroom observations, and LMS log records were subjected to thematic analysis. This approach allowed for the systematic identification and interpretation of patterns within the data, offering rich insights into the EBA tension.

#### 3.3.1 Interview data analysis

The interview recordings were transcribed verbatim and imported into NVivo 14 for systematic organization and analysis. Thematic analysis followed Braun and Clarke’s six-phase process, which included data familiarization, initial coding, theme generation, theme review, theme definition, and final reporting. Coding was conducted both deductively, using predefined categories from Self-Determination Theory and Fredricks et al.’s (2004) engagement model, and inductively, allowing for the emergence of unanticipated themes specific to the EBA construct.

The following eight thematic areas served as a foundation for the coding framework: (1) prior experiences in English learning, (2) use of AI tools during and outside class time, (3) participation strategies in learning activities, (4) emotional and cognitive reactions to AI-supported tasks, (5) motivational goals and driving factors, (6) perceived autonomy and control in the learning process, (7) identity as English learners, and (8) future learning expectations and suggestions. Each area was introduced through open-ended questions, with supplementary prompts used to encourage deeper responses.

Special attention was paid to linguistic markers of motivational detachment, emotional dissonance, and shifts in epistemic stance. Bilingual instances and code-switching patterns were considered analytically relevant, as they often reflected affective positioning or conceptual emphasis. To ensure reliability, a second coder independently reviewed a subset of the data, and any discrepancies were resolved through discussion and consensus.

#### 3.3.2 Classroom observations analysis

Field notes from classroom observations were analyzed thematically, regarding observable indicators of behavioral, emotional, and cognitive engagement. The coding framework was adapted from Fredricks et al. (2004), enabling structured categorization of learner behaviors. Observed dissonance between active classroom participation and signs of emotional disengagement (e.g., passive facial expressions, minimal peer interaction) was documented to identify potential EBA cases.

Triangulation with interview findings facilitated the identification of learners whose surface-level engagement behaviors did not align with their underlying motivational states.

#### 3.3.3 LMS log data analysis

Behavioral data from Xuexitong (学习通), the AI-powered LMS used throughout the two-semester EFL course, were descriptively analyzed. Key metrics included login frequency, time-on-task duration, assignment submission and revision frequency, and interaction with AI-generated feedback. These data provided quantitative indicators of behavioral engagement and served to complement the qualitative findings.

By cross-referencing LMS logs with interview and observation data, learners exhibiting high online engagement, but low self-reported motivation, were identified as prototypical EBA cases. This triangulated approach enhanced the validity of the findings and contributed to a multidimensional understanding of engagement and motivation in AI-mediated learning environments.

Overall, the integration of multiple data sources through thematic analysis supported a nuanced exploration of the EBA phenomenon and its manifestation among EFL learners interacting with generative AI tools.

Following the recommendation of Morse et al. (2002), reliability and validity were addressed through strategies embedded in the research process. To enhance reliability, a second coder independently analyzed 20% of the interview transcripts. Intercoder agreement, measured using Cohen’s Kappa, was 0.82, indicating substantial consistency. Discrepancies were resolved through discussion and agreement.

To minimize researcher bias and support validity, both deductive and inductive coding approaches were used. Thematic categories were informed by established theoretical frameworks, while additional patterns were identified directly from participant responses. Direct quotes were included to support each theme and illustrate how findings were grounded in the data. Detailed descriptions of the learning context and participant characteristics were provided to enhance transferability and allow readers to judge the applicability of the findings to similar contexts.

## 4 Findings

The thematic analysis of the research data revealed three principal categories that, together, delineate a distinctive learner profile marked by high behavioral engagement but limited motivational autonomy—designated here as the EBA profile. These categories—performative participation, motivational stagnation, and identity ambivalence—articulate the complex interplay through which generative AI tools influence learners’ behavioral patterns, cognitive self-regulation, and motivational dynamics and identity reconfiguration in English language acquisition.

### 4.1 Performative participation: engagement as institutional compliance

A central theme identified in participant accounts is the notion of performative engagement, where learners engage with GenAI tools—such as DeepSeek, Doubao (豆包), and Kimi—primarily to fulfill institutional requirements rather than to pursue meaningful learning. These tools are commonly used for routine academic tasks such as grammar correction, sentence translation, and writing assignments. While such use may appear to demonstrate active participation, especially through analytics on learning platforms, closer analysis reveals a surface-level engagement driven by external demands.

When asked, “*Do you learn English because you are interested, or mainly because it’s required*?,” only 6 out of 39 participants cited personal interest or communicative purposes. One remarked, “*I’m personally interested in English*” (Appendix 1–Q1–1), while another shared, “*I have some interest; I want to travel abroad and communicate with foreigners*” (Appendix 1–Q1–2). In contrast, the remaining 33 students indicated extrinsic motivations, linking their learning to institutional goals such as passing exams, gaining credits, or meeting course requirements. As one student noted, “*It’s mainly a school requirement; I’m mostly passive and grade-focused*” (Appendix 1–Q1–3), while another explained, “*The main reason is school requirements—completing assignments and passing tests to earn credits*” (Appendix 1–Q1–4). Additionally, many students reported learning English primarily to prepare for the vocational-to-university transfer examination. As one student shared, “*I mainly study English for the transfer exam*” (Appendix 1–Q1–5), while another echoed, “*For the transfer, I must pass English*” (Appendix 1–Q1–6). These responses further illustrate that students’ engagement with English is largely driven by institutional and exam-related demands rather than by personal interest or intrinsic motivation.

These extrinsic drivers are reflected in classroom behavior. When asked, “How do you behave during AI-assisted English classes? Are you active or passive?,” 25 out of 39 students described themselves as “*passive*” or “*somewhat passive*” (Appendix 1–Q2–1), suggesting a lack of personal agency or enthusiasm. Moreover, in response to the question, “Do you feel more like a learner or a tool user when working with AI?,” only 6 participants identified as learners; the rest viewed themselves more as tool users. One participant reflected, “*I feel more like a tool user and do not take initiative in learning*” (Appendix 1–Q3–1).

These reflections reveal a mismatch between external engagement and internal motivation. While institutional systems may read tool usage as active learning, many students describe their actions as a form of obligation rather than authentic learning. In this sense, GenAI tools become instrumental means to complete academic tasks, often replacing the cognitive effort and intrinsic interest that meaningful learning requires.

### 4.2 Motivational stagnation: cognitive overload as an obstacle

Many participants reported a noticeable decline in their motivation to learn English during college. This reduction was frequently linked to increased academic pressure, the complexity of learning tasks, and persistent struggles with vocabulary and grammar. Although some students acknowledged the importance of English for exams or future job opportunities, these instrumental goals were often overshadowed by emotional fatigue and anxiety—particularly around high-stakes exams such as the CET-4. Several learners also noted that English felt increasingly disconnected from their personal interests or everyday needs.

Compared to their earlier schooling, where teacher support and structured classroom routines occasionally sparked interest, the transition to more self-directed college learning was often described as demotivating. Without sufficient guidance, autonomy led some students to disengage. For example, one remarked, “*Now English is just a task to complete—I do not feel motivated*” (Appendix 2–Q4–1). While a few did experience satisfaction when successfully using GenAI tools, such moments were rare and insufficient to sustain meaningful motivation.

In response to the question, “Has your motivation for learning English changed since using AI tools?,” only 8 out of 39 participants reported increased motivation. One student explained, “*Yes, I feel that learning English is not as difficult anymore*” (Appendix 2–Q4–2). However, 12 students described growing dependence on AI tools rather than increased engagement. As one stated, “*I feel increasingly dependent because AI is too powerful and limits students’ thinking*” (Appendix 2–Q4–3). Thirteen participants reported “*no change*” (Appendix 2–Q4–4) in their motivation, while a few mentioned feeling “*less motivated*” (Appendix 2–Q4–5). One noted, “*After using AI for a while, I do not really want to think by myself*” (Appendix 2–Q4–6).

These reflections suggest that although GenAI is perceived as useful for academic tasks—especially grammar correction and translation—it does not consistently lead to deeper or sustained motivation. One contributing factor appears to be cognitive overload. For students with lower English proficiency, using AI required additional mental effort—choosing tools, understanding complex outputs, and navigating digital interfaces. Rather than simplifying the learning process, this sometimes made it more overwhelming.

This burden was mirrored in students’ emotional responses. While a few described curiosity or brief satisfaction, many reported emotional detachment, using expressions such as “*indifferent*,” “*no feeling*,” “*calm*,” or “*bored*” (Appendix 2–Q4–7). Some also mentioned anxiety or confusion, especially when uncertain about how to use AI effectively. Taken together, these responses highlight that for many learners—especially those with limited digital or language confidence—GenAI tools are seen more as convenient aids than as meaningful sources of engagement or motivation.

### 4.3 Identity ambivalence: GenAI as enabler and eroder

Participants’ reflections revealed a dual perspective on the role of GenAI in shaping their identities as English language learners. On the one hand, many students regarded AI tools as empowering supports that reduced anxiety and increased confidence. Several described becoming more willing to engage in learning tasks. One student shared, “*I’ve become more proactive*” (Appendix 3–Q5–1), while another remarked, “*It made me more confident and active*” (Appendix 3–Q5–2). For these learners, GenAI served as a cognitive scaffold that helped bridge linguistic gaps and supported independent learning, particularly in reading, writing, and comprehension. As a result, GenAI was seen as an enabler that made English learning feel more accessible and less intimidating.

On the other hand, a comparable group of students expressed concerns that overreliance on GenAI was weakening their sense of ownership and engagement. Some reported reduced motivation and a lack of challenge. One participant stated, “*With AI, learning English no longer feels challenging*” (Appendix 3–Q5–3). Others voiced stronger disengagement, as shown in the comment, “*It’s boring; learning English feels useless*” (Appendix 3–Q5–4). In contrast, several students emphasized the need for personal effort, stating, “*Progress depends on oneself and the willingness to learn*” (Appendix 3–Q5–5), and “*Progress relies not only on AI, but also on self-discipline, hard work, and determination*” (Appendix 3–Q5–6). These responses suggest that while GenAI can support learning, it may also discourage reflection and reduce students’ active participation in the learning process.

This ambivalence became more pronounced when participants were asked, “*If AI tools were no longer used in your English class, how would you feel?*” Many described strong negative emotions, including “*anxiety*,” “*loss*,” and “*inconvenience*.” One student commented, “*I would feel anxious because without AI, I truly would not understand anything*” (Appendix 3–Q6–1). Another explained, “*I would feel lost and anxious, as if my work would no longer be complete or perfect*” (Appendix 3–Q6–2). These statements highlight the deep integration of AI into their academic routines. At the same time, a smaller number of students welcomed the potential benefits of AI removal. One reflected, “*I would feel a bit panicked, but it might help me think and work independently*” (Appendix 3–Q6–3), and another shared, “*It would encourage independent thinking and help me improve*” (Appendix 3–Q6–4). A few students were indifferent, saying “*no effect*” *or* “*it does not matter*” (Appendix 3–Q6–5).

In summary, learners’ perspectives on GenAI reveal a complex interplay of dependence and agency. While many appreciate the confidence and convenience it offers, others caution against its potential to limit critical thinking and personal growth. This tension illustrates a broader educational challenge: integrating AI in ways that enhance, rather than replace, learners’ autonomy and identity development.

## 5 Discussion

This study introduces the EBA construct as a new lens for understanding engagement in AI-augmented EFL learning, particularly within the under-researched context of Chinese HVCs. The findings challenge the assumption that frequent use of GenAI tools necessarily enhances student engagement (Nguyen et al., 2024; Li and Chiu, 2025). Although learners showed high levels of behavioral participation, their self-reports revealed a disconnect between outward actions and internal motivation. This discrepancy calls for a reassessment of how engagement is measured (Reeve and Tseng, 2011) and interpreted in AI-mediated education.

This concern is especially relevant in systems that emphasize measurable outcomes. In vocational education, where standardized assessments and credentialing prevail, AI tools may inadvertently promote surface-level interaction rather than deeper learning (Avsheniuk et al., 2025; Sari, 2023). As such, relying solely on digital performance data can be misleading. Instead, assessment frameworks should be expanded to include learners’ constructive motivation, and meaningful learning (Bandura, 2006; Reeve, 2013).

Another insight relates to learner motivation in AI-supported environments. While GenAI tools are often praised for promoting autonomy and engagement (Pan, 2023; Tang, 2025), this study finds that such outcomes are not universal. For less-prepared learners, AI platforms may cause confusion or overload (Sweller, 2011; Jose et al., 2025), particularly when managing multiple tools or interpreting complex feedback. This suggests that motivation may not be caused by AI use but rather shapes how AI is used. Learners with strong self-regulation and agency are more likely to benefit from GenAI, while others may struggle to engage meaningfully (Zheng et al., 2024). Without proper support, these tools can unintentionally demotivate rather than motivate.

The findings also reveal identity challenges in AI-mediated learning. While some students gained confidence, others felt their role in the learning process was diminished, echoing concerns raised by Zhou and Hou (2024) and Liu et al. (2025). Students’ over-reliance on AI-powered conversational systems—especially those using generative models like ChatGPT—can undermine their essential cognitive skills. Despite benefits such as streamlined research and faster task completion, this dependency often leads to reduced critical thinking, weakened decision-making, diminished analytical reasoning, and increased risk of academic dishonesty (like hallucinations, bias, or plagiarism) (Zhai et al., 2024).

The findings of this study have important implications for teaching, technology design, and teacher education. For EFL teachers, it is important to go beyond checking whether students complete tasks. Teachers should include more activities that help students think about their own learning. For example, students can keep weekly journals to reflect on how they feel during learning, what they find useful, or what goals they have. Teachers can also organize small group discussions where students talk about challenges and learning strategies. At the end of each unit, a short discussion led by the teacher can help students connect their feelings and progress. These activities make learning more personal and help reveal students’ real motivation. For AI designers, the results suggest that systems should support motivation, not only performance. One possible strategy is to include short reflective prompts before or after tasks that ask students to think about their effort or interest. AI systems can also use check-in questions during tasks to ask how students feel or how confident they are. In addition, giving students some choice—like choosing the order of tasks or the topic—can help them feel more in control and motivated. Teacher training should also include basic knowledge of AI and its effects on learning. Teachers need to learn how to recognize signs of “Engaged but Amotivated” learners—for example, students who do everything on time but show no interest. Training should help teachers understand how to support students emotionally and use classroom strategies to balance the role of AI.

Overall, this study suggests that successful AI-supported learning must include more than just completing tasks. Motivation, emotional connection, and learner choice are key. The EBA construct helps identify problems that may not be visible through task data alone. This can help teachers, designers, and school leaders make better decisions when using AI in education.

## 6 Conclusion

This study has introduced and explored the concept of EBA learners in AI-enhanced EFL instruction within a Chinese HVC context. Through a qualitative case study design incorporating classroom observations, semi-structured interviews, and LMS data analysis, the research uncovered a nuanced motivational profile: students who engage behaviorally with GenAI tools but lack intrinsic motivation and emotional connection. The findings reveal that while GenAI platforms effectively support task completion and academic performance metrics, they do not necessarily foster authentic motivation or deep learner identity formation. Instead, for many learners—particularly those with low proficiency or digital literacy—AI tools can reinforce passive learning habits, cognitive dependence, emotional disengagement, and motivational decline.

The EBA construct, grounded in Self-Determination Theory and engagement frameworks, provides a critical lens for understanding this phenomenon. It challenges prevalent educational assumptions that equate visible participation with authentic motivation and learning achievement and calls for more robust, multidimensional approaches to assessing learner motivation and engagement. By drawing attention to the affective and motivational consequences of GenAI integration, the study highlights the urgent need to rethink AI’s role in language pedagogy—particularly in educational settings where learners are structurally and motivationally disadvantaged.

Despite its contributions, this study is not without limitations. First, the research was limited to a single higher vocational college in eastern China. Although the site was purposefully selected for its proactive implementation of GenAI tools, its institutional profile and regional context may not fully represent the broader HVC system across China. While the findings are not intended to be statistically generalizable, they may offer analytical insights applicable to similar educational contexts. Future studies should explore whether comparable patterns of EBA emerge in other vocational institutions or even university settings, particularly those in less economically developed areas or with varying levels of digital infrastructure and pedagogical support.

Second, while the study employed multiple qualitative data sources to enhance triangulation and credibility, it relied heavily on self-reported data, which may be influenced by social desirability or limited metacognitive awareness among participants. In particular, students’ ability to articulate motivational states or emotional reactions may be constrained by linguistic limitations or cultural norms that discourage overt expressions of disaffection. Complementary use of quantitative methods—such as validated motivation and engagement scales—could enhance the robustness and generalizability of future investigations.

Lastly, while this study focused on students, the teacher perspective was not systematically examined. Given the crucial mediating role that teachers play in shaping learner experiences with AI, further research should investigate how teacher beliefs, practices, and emotional labor affect students’ engagement and motivation in AI-mediated environments. This dual perspective would provide a more comprehensive understanding of the ecosystem within which EBA manifests.

This study calls for a critical reconsideration of how generative AI is integrated into EFL education. For learners in higher vocational colleges, who already face motivational, structural, and affective barriers, GenAI should not merely serve as a tool for efficiency or compliance, but as a pedagogical partner in cultivating autonomy, meaning-making, and learner identity. Recognizing and addressing the EBA profile is essential for designing equitable, motivationally sustainable AI-enhanced learning environments—ones that do not confuse activity for authenticity, or automation for agency. Only by bridging this gap can we ensure that the promise of AI in education is fully realized in human-centered and inclusive ways.

## Data Availability

The original contributions presented in the study are included in the article/Supplementary material, further inquiries can be directed to the corresponding author.

## References

[ref1] Abed I. N. Al-Tamimi R. S. A. Ghanim K. S. Nashmi B. H. (2024) A qualitative study of English language teachers’ perceptions on teaching English language through artificial intelligence in public education in Iraq. International Conference on Intelligent Systems, Blockchain, and Communication Technologies (125–136). Springer Nature.

[ref2] Ahimsa A. F. H. Khawa D. (2025). The impact of artificial intelligence on English language teaching: opportunities and challenges in technology era. IDEAS 13, 191–201. doi: 10.24256/ideas.v13i1.6270

[ref3] Alasadi E. A. Baiz C. R. (2023). Generative AI in education and research: opportunities, concerns, and solutions. J. Chem. Educ. 100, 2965–2971. doi: 10.1021/acs.jchemed.3c00323

[ref4] Ali J. K. M. Shamsan M. A. A. Hezam T. A. Mohammed A. A. (2023). Impact of ChatGPT on learning motivation: teachers and students’ voices. J. Engl. Stud. Arabia Felix 2, 41–49. doi: 10.56540/jesaf.v2i1.51

[ref5] Aly A. H. Lustyantie N. Chaeruman U. A. (2024). Empowering motivation through AI in teaching English for specific purposes. Int. J. Engl. Comp. Lit. Stud. 5, 1–11. doi: 10.47631/ijecls.v5i6.863

[ref6] Aspers P. Corte U. (2019). What is qualitative in qualitative research? Qual. Sociol. 42, 139–160. doi: 10.1007/s11133-019-9413-7, PMID: 31105362 PMC6494783

[ref7] Avsheniuk N. Seminikhyna N. Ruban L. Sviatiuk Y. (2025). Exploring overreliance on AI tools in English for specific purposes courses: challenges and implications for learning and academic integrity. Arab World English J. 2025, 3–20. doi: 10.24093/awej/AI.1

[ref8] Bandura A. (2006). Toward a psychology of human agency. Perspect. Psychol. Sci. 1, 164–180. doi: 10.1111/j.1745-6916.2006.00011.x, PMID: 26151469

[ref9] Banun F. F. (2025). The use of AI in increasing motivation to learn English for university students. Educ. Praxis J. 1, 1–7. doi: 10.32529/epj.v1i1.3782

[ref10] Baskara F. R. (2023). Integrating chatGPT into EFL writing instruction: benefits and challenges. Int. J. Educ. Learn. 5, 44–55. doi: 10.31763/ijele.v5i1.858

[ref11] Bhatt P. Muduli A. (2024). AI learning intention, learning engagement and behavioral outcomes: an empirical study. J. Manage. Dev. 43, 920–938. doi: 10.1108/JMD-05-2024-0173

[ref12] Biggs J. (1988). The role of metacognition in enhancing learning. Aust. J. Educ. 32, 127–138. doi: 10.1177/000494418803200201

[ref13] Cao A. E. Chen F. (2023). Low-resource language based cognitive psychology in English language analysis: a state-of-art for China’s higher vocational colleges. ACM Trans. Asian Low Resour. Lang. Inf. Process. 1–14. doi: 10.1145/3588768

[ref14] Cheng C. Y. (2020). “Knowledge and virtues: Confucian education as life education and its modern relevance” in Confucian perspectives on learning and self-transformation: International and cross-disciplinary approaches. eds. Reichenbach R. Kwak D.-J. (Cham: Springer), 27–43.

[ref15] Clarke V. Braun V. (2017). Thematic analysis. J. Posit. Psychol. 12, 297–298. doi: 10.1080/17439760.2016.1262613

[ref16] Crompton H. Edmett A. Ichaporia N. Burke D. (2024). AI and English language teaching: affordances and challenges. Br. J. Educ. Technol. 55, 2503–2529. doi: 10.1111/bjet.13460

[ref17] Deci E. L. Ryan R. M. (1985). Intrinsic motivation and self-determination in human behavior. New York: Springer.

[ref18] Deci E. L. Ryan R. M. (2000). Self-determination theory and the facilitation of intrinsic motivation, social development, and well-being. Am. Psychol. 55, 68–78. doi: 10.1037/0003-066X.55.1.68, PMID: 11392867

[ref19] Denzin N. K. Lincoln Y. S. (2008). “Introduction: the discipline and practice of qualitative research” in Strategies of qualitative inquiry. eds. Denzin N. K. Lincoln Y. S.. 3rd ed (California: Sage Publications, Inc), 1–43.

[ref20] Ebadi S. Amini A. (2024). Examining the roles of social presence and human-likeness on Iranian EFL learners’ motivation using artificial intelligence technology: a case of CSIEC chatbot. Interact. Learn. Environ. 32, 655–673. doi: 10.1080/10494820.2022.2096638

[ref21] Ellikkal A. Rajamohan S. (2024). AI-enabled personalized learning: empowering management students for improving engagement and academic performance. Vilakshan XIMB J. Manag. 21, 1–17. doi: 10.1108/XJM-02-2024-0023

[ref22] Elliot A. J. (1999). Approach and avoidance motivation and achievement goals. Educ. Psychol. 34, 169–189. doi: 10.1207/s15326985ep3403_3

[ref23] Elliot A. J. Moller A. C. (2003). Performance-approach goals: good or bad forms of regulation? Int. J. Educ. Res. 39, 339–356. doi: 10.1016/j.ijer.2004.06.003

[ref24] Elliot A. J. Shell M. M. Henry K. B. Maier M. A. (2005). Achievement goals, performance contingencies, and performance attainment: an experimental test. J. Educ. Psychol. 97, 630–640. doi: 10.1037/0022-0663.97.4.630

[ref25] Fandiño F. G. E. Muñoz L. D. Velandia A. J. S. (2019). Motivation and e-learning English as a foreign language: a qualitative study. Heliyon 5:e02394. doi: 10.1016/j.heliyon.2019.e02394, PMID: 31528742 PMC6742856

[ref26] Fathi J. Rahimi M. (2024). Utilising artificial intelligence-enhanced writing mediation to develop academic writing skills in EFL learners: a qualitative study. Comput. Assist. Lang. Learn. 2024, 1–40. doi: 10.1080/09588221.2024.2374772

[ref27] Febriani H. (2024). Navigating English learning with AI: a qualitative study of university students’ experiences. PPSDP Int. J. Educ. 3, 220–232. doi: 10.59175/pijed.v3i2.309

[ref28] Fitria T. N. (2023). Artificial intelligence (AI) technology in OpenAI ChatGPT application: a review of ChatGPT in writing English essay. ELT Forum 12, 44–58. doi: 10.15294/elt.v12i1.64069

[ref29] Fredricks J. A. Blumenfeld P. C. Paris A. H. (2004). School engagement: potential of the concept, state of the evidence. Rev. Educ. Res. 74, 59–109. doi: 10.3102/00346543074001059

[ref30] Gilbert K. M. Elliot A. J. (2024). Metamotivational task knowledge of performance-approach and performance-avoidance achievement goals. Curr. Psychol. 43, 32288–32302. doi: 10.1007/s12144-024-06773-0

[ref31] Gong L. Deng H. (2024) Impact of AI technologies on education modernization in China: A knowledge graph analysis. In Proceedings of the 2024 International Conference on Computer and Multimedia Technology (64–74).

[ref32] Hao L. J. Tian K. Leng C. H. Salleh U. K. B. M. (2024). The mediating effects of critical thinking on the motivation and creativity of business English learners in the age of AI: cognitive flexibility theory. Think. Skills Creat. 53, 1–13. doi: 10.1016/j.tsc.2024.101578

[ref33] He Y. (2024). The metaphor of AI in writing in English: a reflection on EFL learners’ motivation to write, enjoyment of writing, academic buoyancy, and academic success in writing. Int. Rev. Res. Open Distrib. Learn. 25, 271–286. doi: 10.19173/irrodl.v25i3.7769

[ref34] Hong W. C. H. (2023). The impact of ChatGPT on foreign language teaching and learning: opportunities in education and research. J. Educ. Technol. Innov. 5, 37–45. doi: 10.61414/jeti.v5i1.103

[ref35] Huang Q. Y. Li W. L. Z Y. M. (2025). Enhancing deep learning and motivation in university English education through AI technology: a quasi-experimental study. Asian J. Educ. Soc. Stud. 51, 452–463. doi: 10.9734/ajess/2025/v51i41883

[ref36] Huang J. Tan M. (2023). The role of ChatGPT in scientific communication: writing better scientific review articles. Am. J. Cancer Res. 13, 1148–1154. 37168339 PMC10164801

[ref37] Jiang R. (2022). How does artificial intelligence empower EFL teaching and learning nowadays? A review on artificial intelligence in the EFL context. Front. Psychol. 13, 1–8. doi: 10.3389/fpsyg.2022.1049401. doi: 10.3389/fpsyg.2022.1049401 PMC9709327 36467167

[ref38] Jose B. Cherian J. Verghis A. M. Varghise S. M. S M. Joseph S. (2025). The cognitive paradox of AI in education: between enhancement and erosion. Front. Psychol. 16:1550621. doi: 10.3389/fpsyg.2025.1550621, PMID: 40297599 PMC12036037

[ref39] Kayaalp Z. (2025). “The impact of AI-blended learning on EFL students’ English language proficiency, attitudes, and motivation” in English studies: A multifaceted lens. eds. Özkan Y. Kara M. Dinçer A. Sucak D. Harmandar B. B. Deniz E.. (Ankara: Black Swan Publishing House), 403–424.

[ref40] Li Y. Chiu T. K. (2025). The mediating effects of needs satisfaction on the relationship between teacher support and student engagement with generative artificial intelligence (GenAI) chatbots from a self-determination theory (SDT) perspective. Educ. Inf. Technol., 1–20. doi: 10.1007/s10639-025-13574-w

[ref41] Liando N. V. F. Tatipang D. P. Rorimpandey R. Kumayas T. Saudah K. Iskandar I. (2025). AI-powered language learning: a blessing or a curse for English language education? Stud. Engl. Lang. Educ. 12, 301–311. doi: 10.24815/siele.v12i1.34842

[ref42] Liu G. L. Darvin R. Ma C. (2024a). Exploring AI-mediated informal digital learning of English (AI-IDLE): a mixed-method investigation of Chinese EFL learners’ AI adoption and experiences. Comput. Assist. Lang. Learn., 1–29. doi: 10.1080/09588221.2024.2310288

[ref43] Liu G. L. Darvin R. Ma C. (2024b). Unpacking the role of motivation and enjoyment in AI-mediated informal digital learning of English (AI-IDLE): a mixed-method investigation in the Chinese context. Comput. Hum. Behav. 160:108362. doi: 10.1016/j.chb.2024.108362

[ref44] Liu Y. Quan Q. (2022). AI recognition method of pronunciation errors in oral English speech with the help of big data for personalized learning. J. Inf. Knowl. Manag. 21:2240028. doi: 10.1142/S0219649222400287

[ref45] Liu W. Wang Y. (2024). The effects of using AI tools on critical thinking in English literature classes among EFL learners: an intervention study. Eur. J. Educ. 59:e12804. doi: 10.1111/ejed.12804

[ref46] Liu X. Xiao Y. (2025). Chinese university teachers’ engagement with generative AI in different stages of foreign language teaching: a qualitative enquiry through the prism of ADDIE. Educ. Inf. Technol. 30, 485–508. doi: 10.1007/s10639-024-13117-9

[ref47] Liu G. L. Zou M. M. Soyoof A. Chiu M. M. (2025). Untangling the relationship between AI-mediated informal digital learning of English (AI-IDLE), foreign language enjoyment and the ideal L2 self: evidence from Chinese university EFL students. Eur. J. Educ. 60:e12846. doi: 10.1111/ejed.12846

[ref48] Luo M. Hu X. Zhong C. (2025). The collaboration of AI and teacher in feedback provision and its impact on EFL learner’s argumentative writing. Educ. Inf. Technol. 30, 17695–17715. doi: 10.1007/s10639-025-13488-7, PMID: 40832454

[ref49] Madinabonu J. (2024). Methodological foundations of using the possibilities of artificial intelligence in the development of motivation to learn English among students. Western Europ. J. Linguist. Educ. 2, 40–42.

[ref50] Ministry of Education of the People’s Republic of China (2024) 2024 National education development statistics bulletin. Available online at: http://www.moe.gov.cn/jyb_sjzl/sjzl_fztjgb/202410/t20241024_1159002.html (Accessed March 16, 2025)

[ref51] Morse J. M. Barrett M. Mayan M. Olson K. Spiers J. (2002). Verification strategies for establishing reliability and validity in qualitative research. Int J Qual Methods 1, 13–22. doi: 10.1177/160940690200100202

[ref52] Moybeka A. M. Syariatin N. Tatipang D. P. Mushthoza D. A. Dewi N. P. J. L. Tineh S. (2023). Artificial intelligence and English classroom: the implications of AI toward EFL students’ motivation. Edumaspul 7, 2444–2454. doi: 10.33487/EDUMASPUL.V7I2.6669

[ref53] Nguyen A. Kremantzis M. Essien A. Petrounias I. Hosseini S. (2024). Enhancing student engagement through artificial intelligence (AI): understanding the basics, opportunities, and challenges. J. Univ. Teach. Learn. Pract. 21, 1–13. doi: 10.53761/caraaq92

[ref54] Özdere M. (2023). The integration of artificial intelligence in English education: opportunities and challenges. Lang. Educ. Technol. 3, 137–172.

[ref55] Pan X. (2023). Online learning environments, learners’ empowerment, and learning behavioral engagement: the mediating role of learning motivation. SAGE Open 13, 1–16. doi: 10.1177/21582440231205098

[ref56] Pan Q. Dapat R. O. (2023). Evaluation of higher vocational English curriculum toward enhanced program implementation. J. Educ. Educ. Res. 5, 6–10. doi: 10.54097/jeer.v5i1.11548

[ref560] Pan Z. Wang Y. (2025). From technology‐challenged teachers to empowered digitalized citizens: exploring the profiles and antecedents of teacher ai literacy in the Chinese EFL context. Euro. J. Educ. 60:e70020. doi: 10.1111/ejed.70020

[ref57] Pido N. W. T. Machmud K. Ibrahim M. S. Miolo S. Hasanuddin C. (2025). Challenges of using artificial intelligence (AI) in English productive skills learning: a thematic analysis of the student experience. Int. J. Soc. Sci. Hum. Res. 8, 2350–2354. doi: 10.5281/zenodo.15251138

[ref58] Ramadhani A. (2025). Exploring the role of AI-based conversation in enhancing student motivation for English-speaking practice. Tadangate J. Educ. Res. 2, 25–36.

[ref59] Reeve J. (2013). How students create motivationally supportive learning environments for themselves: the concept of agentic engagement. J. Educ. Psychol. 105, 579–595. doi: 10.1037/a0032690

[ref60] Reeve J. Tseng C. M. (2011). Agency as a fourth aspect of students’ engagement during learning activities. Contemp. Educ. Psychol. 36, 257–267. doi: 10.1016/j.cedpsych.2011.05.002

[ref61] Riser P. (2025). Generative artificial intelligence (AI) in the high school English classroom and its effect on students’ identity and teachers’ practice. Engl. Educ. 59, 176–192. doi: 10.1080/04250494.2025.2491361

[ref62] Ryan R. M. Deci E. L. (2020). Intrinsic and extrinsic motivation from a self-determination theory perspective: definitions, theory, practices, and future directions. Contemp. Educ. Psychol. 61:101860. doi: 10.1016/j.cedpsych.2020.101860

[ref63] Sari N. (2023). The role of artificial intelligence (AI) in developing English language learner’s communication skills. J. Educ. 6, 750–757. doi: 10.31004/joe.v6i1.2990

[ref64] Shen H. Ye X. Zhang J. Huang D. (2024). Investigating the role of perceived emotional support in predicting learners’ well-being and engagement mediated by motivation from a self-determination theory framework. Learn. Motiv. 86:101968. doi: 10.1016/j.lmot.2024.101968

[ref65] Silitonga L. M. Hawanti S. Aziez F. Furqon M. Zain D. S. M. Anjarani S.. (2023). The impact of AI chatbot-based learning on students’ motivation in English writing classroom. In International Conference on Innovative Technologies and Learning (pp. 542–549). Springer.

[ref66] Skinner E. A. (2016). “Engagement and disaffection as central to processes of motivational resilience and development” in Handbook of motivation at school. eds. Wentzel K. R. Miele D. B.. 2nd ed (New York: Routledge), 145–168.

[ref67] Suciati S. Silitonga L. M. Wiyaka C. Huang C. Y. Anggara A. A. (2024). Enhancing engagement and motivation in English writing through AI: the impact of ChatGPT-supported collaborative learning. In International Conference on Innovative Technologies and Learning (pp. 205–214). Springer.

[ref68] Sweller J. (2011). “Cognitive load theory” in Psychology of learning and motivation. ed. Ross B. H., vol. 55 (San Diego: Academic Press), 37–76.

[ref69] Tang X. (2025). L2 writing with AI: perceptions and engagement of EFL learners in China. Engl. Lang. Teach. 18, 68–75. doi: 10.5539/elt.v18n2p68

[ref70] Tran M. (2024) Enhancement of performance and motivation through the application of AI and robots in an English class. In eLearn: World Conference on EdTech (671–683). AACE.

[ref71] Waluyo B. Kusumastuti S. (2024). Generative AI in student English learning in Thai higher education: more engagement, better outcomes? Soc. Sci. Humanit. Open 10:101146. doi: 10.1016/j.ssaho.2024.101146

[ref72] Wang W. (2024) Exploring the attitudes of higher vocational college students toward artificial intelligence: A case study in China. In 2024 9th International Conference on Modern Management, Education and Social Sciences (pp. 593–604). Atlantis Press.

[ref73] Wang Z. Chen Q. (2021). Understanding challenges for Chinese vocational college students in improving writing skills. Int. J. Learn. Teach. 7, 272–277. doi: 10.18178/ijlt.7.4.272-277

[ref74] Wang X. Gao Y. Wang Q. Zhang P. (2025). Fostering engagement in AI-mediated Chinese EFL classrooms: the role of classroom climate, AI literacy, and resilience. Eur. J. Educ. 60:e12874. doi: 10.1111/ejed.12874

[ref75] Wang F. Zhou X. Li K. Cheung A. C. Tian M. (2025). The effects of artificial intelligence-based interactive scaffolding on secondary students’ speaking performance, goal setting, self-evaluation, and motivation in informal digital learning of English. Interact. Learn. Environ., 1–20. doi: 10.1080/10494820.2025.2470319

[ref76] Wei L. (2023). Artificial intelligence in language instruction: impact on English learning achievement, L2 motivation, and self-regulated learning. Front. Psychol. 14:1261955. doi: 10.3389/fpsyg.2023.1261955, PMID: 38023040 PMC10658009

[ref77] Wei Q. Wang J. Shen D. (2025). A technical communication course design for Chinese higher vocational English majors. Educ. Rev. USA, 9, 232–238. doi: 10.26855/er.2025.02.012

[ref78] Wei W. Zhao A. Ma H. (2025). Understanding how AI chatbots influence EFL learners’ oral English learning motivation and outcomes: evidence from Chinese learners. IEEE Access 13, 56699–56716. doi: 10.1109/ACCESS.2025.3554545

[ref79] Xiong J. (2011). Understanding higher vocational education in China: vocationalism vs Confucianism. Front. Educ. China 6, 495–520. doi: 10.1007/s11516-011-0143-1

[ref80] Xu Y. (2025). Research on higher vocational Chinese teaching system based on deep neural network. Int. J. High Speed Electron. Syst.:2540455. doi: 10.1142/S0129156425404553

[ref81] Xu J. F. Li J. (2024). Exploring self-regulated learning: a qualitative study of graduate students majoring in English in a human-AI interactive language-learning environment. J. Beijing Int. Stud. Univ. 46, 15–29. doi: 10.12002/j.bisu.550

[ref82] Xu J. Liu Q. (2025). Uncurtaining windows of motivation, enjoyment, critical thinking, and autonomy in AI-integrated education: Duolingo vs. ChatGPT. Learn. Motiv. 89, 1–14. doi: 10.1016/j.lmot.2025.102100

[ref83] Yang J. The perception of pre-service English teachers’ use of AI translation tools in EFL writing. J. Converg. Cult. Technol., 10(1), 121–128. doi: 10.17703/JCCT.2024.10.1.121 (2024).

[ref84] Yang T. (2024). Impact of artificial intelligence software on English learning motivation and achievement. SHS Web Conf. 193:02011. doi: 10.1051/shsconf/202419302011

[ref85] Yaşar H. Karagücük V. (2024). Exploring the relationship between artificial intelligence literacy and English language learning motivation. Int. J. Lang. Educ. Teach. 12, 107–124. doi: 10.71084/ijlet.1561914

[ref86] Yuan L. Liu X. (2025). The effect of artificial intelligence tools on EFL learners’ engagement, enjoyment, and motivation. Comput. Human Behav. 162:108474. doi: 10.1016/j.chb.2024.108474

[ref87] Zhai C. Wibowo S. Li L. D. (2024). The effects of over-reliance on AI dialogue systems on students’ cognitive abilities: a systematic review. Smart Learn. Environ. 11:28. doi: 10.1186/s40561-024-00316-7

[ref88] Zheng Y. Wang Y. Liu K. S. X. Jiang M. Y. C. (2024). Examining the moderating effect of motivation on technology acceptance of generative AI for English as a foreign language learning. Educ. Inf. Technol. 29, 23547–23575. doi: 10.1007/s10639-024-12763-3

[ref740] Zou B. Guan X. Shao Y. Chen P. (2023). Supporting speaking practice by social network-based interaction in artificial intelligence (AI)-assisted language learning. Sustain. 15:2872. doi: 10.3390/su15042872

[ref860] Zhou C. Hou F. (2024). Can AI empower L2 education? Exploring its influence on the behavioural, cognitive and emotional engagement of EFL teachers and language learners. Euro. J. Educ. 59:e12750. doi: 10.1111/ejed.12750

