# Supplementary material for: EBA (Engaged but Amotivated) in AI-enhanced EFL learning: a qualitative study from a Chinese higher vocational context
Source: Front Psychol. 2025 Sep 9;16:1643653. doi: 10.3389/fpsyg.2025.1643653 (PMC12454334; doi:10.3389/fpsyg.2025.1643653)
Supplement: Supplementary file 1 [file Supplementary_file_1.docx]

**Appendix**

This appendix provides the original Chinese transcripts of participant quotes referenced in the Findings section (Section 4). Quotes are organized by subsection and presented in their verbatim form, with corresponding English translations for clarity. These transcripts are included as supplementary material to preserve the authenticity of respondent voices.

1. **Performative Participation: Engagement as Institutional Compliance**

**Prompt (Q1)***: Do you learn English because you’re interested, or mainly because it’s required?*

1. CN: “自己对英语感兴趣。”
   EN:“I’m personally interested in English.”
2. CN: “有点兴趣吧，想去外国旅游，跟外国友人交流。”
   EN: “I have some interest; I want to travel abroad and communicate with foreigners.”
3. CN: “目前是学校要求，为了成绩被动比较多。”
   EN: “It’s mainly a school requirement; I’m mostly passive and grade-focused.”
4. CN: “大部分原因是学校要求，为了完成作业考试拿学分。”
   EN: “The main reason is school requirements—completing assignments and passing tests to earn credits.”
5. CN: “我主要是为了转本考试学英语。”
   EN: “I mainly study English for the transfer exam.”
6. CN: “为了转本，英语是必须要过的。”
   EN: “For the transfer, I must pass English.”

**Prompt (Q2)***: How do you behave during AI-assisted English classes—active or passive?*

1. CN: “被动 / 比较被动。”
   EN: “Passive / somewhat passive.”

**Prompt (Q3)***: Do you feel more like a learner or a tool user when working with AI?*

1. CN: “更像是工具的使用者，不太会主动去学。”
   EN: “I feel more like a tool user and don’t take initiative in learning.”
2. **Motivational Stagnation: Cognitive Overload as an Obstacle**

**Prompt (Q4)***: How has your motivation to learn English changed since using AI tools?*

1. CN: “现在英语就是完成任务，没啥动力。”
   EN: “Now English is just a task to complete—I don’t feel motivated.”
2. CN: “有，觉得学英语没有那么困难了。”
   EN: “Yes, I feel that learning English is not as difficult anymore.”
3. CN: “我感觉越来越依赖，因为AI的功能太过于强大，限制学生的思考。”
   EN: “I feel increasingly dependent because AI is too powerful and limits students’ thinking.”
4. CN: “没有变化。”
   EN: “No change.”
5. CN: “动力减弱。”
   EN: “Less motivated.”
6. CN: “用AI久了，好像不太想自己思考了。”
   EN: “After using AI for a while, I don’t really want to think by myself.”
7. CN: “无感 / 没什么感觉 / 平静 / 无聊。”
   EN: “Indifferent / no particular feeling / calm / bored.”
8. **Identity Ambivalence: GenAI as Enabler and Eroder**

**Prompt (Q5)***: In what ways, if any, has AI changed how you see yourself as an English learner?*

1. CN: “变得更主动吧。”
   EN: “I’ve become more proactive.”
2. CN: “让我变得更自信更主动。”
   EN: “It made me more confident and active.”
3. CN: “使用AI我觉得学英语变得没有挑战性了。”
   EN: “With AI, learning English no longer feels challenging.”
4. CN: “无聊，学英语没用。”
   EN: “It’s boring; learning English feels useless.”
5. CN: “靠自己，想学才能进步。”
   EN: “Progress depends on oneself and the willingness to learn.”
6. CN: “进步不止是靠AI，我觉得还是要靠自己的自觉、努力和坚定。”
   EN: “Progress relies not only on AI, but also on self-discipline, hard work, and determination.”

**Prompt (Q6)***: If AI tools were no longer used in your English class, how would you feel?*

1. CN: “我会焦虑，因为不用AI我就真的一点点都听不懂了。”
   EN: “I would feel anxious because without AI, I truly wouldn’t understand anything.”
2. CN: “失落，焦虑，感觉自己完成得不够完美。”
   EN: “I would feel lost and anxious, as if my work would no longer be complete or perfect.”
3. CN: “感觉还是比较慌乱的，但是也能促进自己独立思考，独立完成。”
   EN: “I would feel a bit panicked, but it might help me think and work independently.”
4. CN: “会促进自己的独立思考，能够更好进步。”
   EN: “It would encourage independent thinking and help me improve.”
5. CN: “没什么影响 / 无所谓。”
   EN: “No effect / it doesn’t matter.”
