# Supplementary material for: EBA (Engaged but Amotivated) in AI-enhanced EFL learning: a qualitative study from a Chinese higher vocational context
Source: Front Psychol. 2025 Sep 9;16:1643653. doi: 10.3389/fpsyg.2025.1643653 (PMC12454334; doi:10.3389/fpsyg.2025.1643653)
Supplement: Supplementary file 2 [file Table_1.docx]

Semi-Structured Interview (Bilingual) anonymous （匿名）

Target Participants: China’s Higher Vocational College EFL Students
Purpose: To explore student engagement, motivation, and AI tool usage in EFL contexts.
Instructions: Conduct interviews in Mandarin. Use follow-up probes where appropriate.

你是否悄悄召唤过AI小助手，让它帮你背单词、改作文、甚至用超自然力量翻译“土味情话”？嗯嗯懂的自然都懂。现在我们的AI学术小队正在召集各路“AI使用者”，开启一场关于英语学习+AI奇遇的访谈冒险！不用打怪、不扣血，全程匿名保护你的小马甲。中文？英文？中英混搭？统统OK！快来投递你的真实经历和吐槽，让我们一起解锁“AI辅学”的隐藏成就，为未来英语学习Buff加满！

# Section 1: Warm-Up & Learning Background (了解背景)

1. Can you tell me a bit about your English learning experience before college?( 在你上大学之前，你是咋学习英语的？可以简单说说你啥时候开始学英语、在哪学的、用过什么方法、有没有参加课外补习班、有没有啥特别的经历或故事、对英语有没有特别喜欢或讨厌的阶段)
2. How do you feel about learning English now? Has your attitude changed since entering college? (你目前对学英语的看法和感受是啥？比如觉得英语难不难、有没有兴趣、学起来轻松还是有压力、有没有动力等。和你上大学之前相比，你对英语学习的态度有没有变化？比如以前很喜欢，现在觉得没意思，或者以前觉得英语很难，现在有信心了，等等。)

# Section 2: In- and Out- Classroom Experience with AI (课 内外中使用AI的体验)

1. What AI tools have you used in your English classes? How do you usually use them? (你在英语课上用过哪些人工智能工具？你通常用这些工具来做什么？比如查单词、翻译句子、写作辅助、语法检查、练口语、批改作文、写作业、回答问题等。)
2. Do you use these AI tools outside of class as well? If so, for what tasks?(在课堂之外，你还会不会用这些AI工具？比如在图书馆，宿舍用 AI做作业、复习、准备考试等。如果会，主要用它们来做哪些事情？比如写作文、查资料、翻译、背单词、练口语、做笔记、和AI聊天练习英语口语等。)
3. What kind of feedback do you receive from AI tools? How do you respond to that feedback? (AI工具通常会给你什么样的反馈？比如：纠正你的语法错误，帮你润色句子，给你写作建议，给你正确答案，或者只是简单的翻译。你是怎么对待AI给你的这些反馈的？你会认真看、会采纳建议、会对比自己的答案、会产生怀疑、完全依赖，还是有时会忽略？你觉得这些反馈对你有没有帮助？)

# Section 3: Behavioral Engagement (行为参与)

1. Do you usually complete tasks that involve AI tools? Why or why not? (你平时做的学习任务，比如英语课后作业、英语课堂练习、英语项目等，是不是经常需要用到AI工具？为什么需要或者不需要用AI工具来完成这些任务？)
2. How do you behave during AI-assisted English classes? Are you active or passive? (当老师允许或鼓励大家在英语课上用AI工具时，你上课时的表现是很主动积极，还是比较被动？“积极”指的是你会主动举手、积极参与讨论、主动用AI工具帮助学习，经常提问或者探索新的知识学习。被动”指的是你只是完成老师布置的任务，很少主动思考和提问。)
3. When you interact with AI tools, do you do it carefully or just try to finish quickly? (你在用AI工具做作业或完成学习任务时，是非常认真仔细地思考、检查、确保质量，还是只是想尽快把任务做完就好？这里“认真”指的是你会花时间理解内容、反复检查、主动学习；尽快做完”指的是你只想完成任务，不太在乎过程和结果，只是一味求快，重在速度。)

# Section 4: Emotional & Cognitive Engagement (情感与认知参与)

1. How do you feel emotionally when using AI in your English class? (当你在英语课上使用AI工具：比如 DeepSeek，豆包，腾讯元宝，ChatGPT，等学习的时候，你心里的感受和情绪是什么？是开心、激动、好奇、无聊、焦虑、害怕、失落，还是别的感觉？)
2. Do you think deeply about the content when using AI tools, or do you mostly follow instructions? (用AI工具学习时，你是主动在思考和理解AI给你的内容，还是只是跟着AI的步骤做，不太动脑筋？“”主动、认真思考”指的是你会花时间理解学习内容，思考背后的原理，尝试举一反三。“机械、被动按照指令”指的是你只是一步步照做，AI让你填什么你就填什么，完成任务为主，不太去深究内容本身。)
3. Can you describe a moment when AI tools helped you understand something better? (你能不能举一个具体的例子，说说有哪一次AI工具真的帮你弄懂了英语学习中的某个知识点？比如AI帮你解释了一个语法点、单词用法、写作结构，或者用AI生成的例句让你突然明白了某个问题。)

# Section 5: Motivation and Purpose (动机与学习目标)

1. What motivates you to participate in English learning activities involving AI? （是什么原因或者动力让你愿意在英语学习中使用AI工具？换句话说：你为什么会用AI来学习英语？是什么推动、促使你用AI来完成学习任务、练习或活动？）
2. Do you learn English because you're interested, or mainly because it's required? （你学习英语的主要原因是什么？是自己对英语有兴趣、觉得学英语有趣、有用、有成就感，所以主动去学？还是主要因为老师或学校要求，比如要完成作业、通过考试、拿学分，不得不学？）
3. Has your motivation for learning English changed since using AI tools? How? （在你开始用AI工具以后，你学习英语的动力有没有发生变化？如果有，是变得更有动力，更愿意学了，还是变得更依赖AI、动力减弱了、觉得学习英语没劲了？？请具体说说变化的内容和原因。）

# Section 6: Autonomy and Control (自主性与控制感)

1. Do you feel you can make choices about how to use AI tools in your learning? （在你的英语学习过程中，你觉得自己能不能自主决定、自己选择如何使用AI工具？也就是说，你有没有“自主权/选择权”去决定AI工具的使用方式？）
2. When AI gives you suggestions or answers, do you always follow them, or do you think for yourself? （当AI工具给你答案或建议时，你是直接按照AI说的去做，还是会结合自己的思考、判断、甚至查资料，然后再决定要不要接受AI的建议？）

# Section 7: Identity and Role as Learner (学习者身份认同)

1. Do you feel more like a learner or a tool user when working with AI? Why? （当你用AI工具学习英语的时候，你觉得自己更像一个“真正的学习者”，主动思考、吸收知识，还是更像一个“工具使用者”，只是把AI当成完成任务的工具，不太投入学习？为什么会有这种感觉？是因为你觉得AI能帮你深入理解内容，还是觉得自己只是机械地利用AI完成任务？）
2. Has AI changed how you see yourself as an English learner? In what way? （你可以想想，AI的使用有没有让你变得更自信、更主动、更依赖，还是更被动？是不是觉得英语学习不再那么难了，抑或是觉得学英语变得无聊/没有挑战性了？甚至觉得英语学习失去意义了，因为自己永远没有 AI 优秀？AI 能够更好完成所有英语任务：写作，翻译，阅读，口语等。你是否更重视用AI解决问题，还是觉得自己进步主要靠AI而不是自己？）

# Section 8: Future Outlook & Reflections (未来展望与反馈)

1. If AI tools were no longer used in your English class, how would you feel? （如果以后英语课不能再用AI工具，你的内心会有什么感受、想法或反应？你会觉得很失落、焦虑、慌乱，不方便，还是觉得没什么影响，甚至觉得反而能促进自己独立思考、独立完成、更好进步？）
2. What advice would you give your teacher about using AI in English classes? （你希望老师在英语课堂上怎么用AI工具，或者怎么引导学生更好地使用AI？比如哪些任务适合用AI辅助完成，哪些部分应该要求学生上交手机完后自己完成。你觉得老师在AI教学方面有哪些做得好的地方，或者有哪些可以改进的地方？）
